# Results of the Telemedicine Program for implementation of the Surviving Sepsis Campaign Protocol in a community Brazilian hospital

**DOI:** 10.1186/cc13207

**Published:** 2014-03-17

**Authors:** C Abreu Filho, M Steinman, A Andrade, F Piza, A Caluza, J Teixeira, M Bracco, R Abaladejo, E Silva

**Affiliations:** 1 Hospital Israelita Albert Einstein, Sao Paulo, Brazil; 2 Hospital Municipal Dr.Moysés Deutsch, Sao Paulo, Brazil

## Introduction

Sepsis has high incidence around the world, approximately 400,000 new cases occur annually in Brazil. In developing countries, it is still an important cause of death due to poor adherence to best- practice medicine protocols. The Brazilian mortality rate of septic shock is around 60%. The aim of this study is to describe the initial results for the implementation of the Brazilian initiative of a Telemedicine (TM) Project for therapeutic support of septic patients in a community hospital in Sao Paulo, Brazil.

## Methods

Since May 2012, a TM Program has been implemented at two hospitals in Sao Paulo - Hospital Municipal Dr. Moysés Deutsch (HMMD), a public, secondary hospital, and Hospital Israelita Albert Einstein (HIAE), a tertiary private philanthropic entity - due to a partnership with Brazilian Health Ministry. A TM Central Command was located at HIAE with Endpoint 97 MXP Cisco^® ^Solution and Medigraf Gowireless^® ^technology. Mobile Intern MXP ISDN/IP Cisco^® ^and Medigraf Gowireless^® ^for the remote hospital (HMMD) via a dedicated connection. At HMMD, the sepsis protocol, based on the Surviving Sepsis Campaign, has been started for every recruited patient admitted to the emergency department (ED) or the ICU, and assessed by the Central Command through TM with an experienced consultant of HIAE. We compared the results of this group of patients with the group of patients with diagnosis of severe sepsis and septic shock, admitted to HMMD during 2011, who were not assisted with TM resources.

## Results

From January to December 2011, 283 septic patients were treated at HMMD, 161 (56.8%) patients were male, mean age was 53.7 years and the hospital mortality was 65.3%. After the implementation of the TM Program, 189 septic patients were admitted to the hospital and evaluated by skilled doctors of HIAE via TM, with the institution of the Surviving Sepsis Campaign protocol. In total, 68.9% of the consultations originated from the ICU and 31.1% from the ED; 111 patients were male (58.4%), mean age was 54.1 years and the hospital mortality was 32.9% (*P *< 0.05).

## Conclusion

Use of the TM Program for the implementation of the Surviving Sepsis Campaign Protocol at a community Brazilian hospital improved compliance with recommended care bundles and significantly decreased the hospital mortality of septic patients.

